# Gender Differences in Global Functional Connectivity During Facial Emotion Processing: A Visual MMN Study

**DOI:** 10.3389/fnbeh.2018.00220

**Published:** 2018-09-25

**Authors:** Jian Zhang, Xiaonan Dong, Luyao Wang, Lun Zhao, Zizheng Weng, Tianyu Zhang, Junyu Sui, Ritsu Go, Qiang Huang, Jinglong Wu, Tianyi Yan

**Affiliations:** ^1^School of Mechatronical Engineering, Intelligent Robotics Institute, Beijing Institute of Technology, Beijing, China; ^2^School of Life Science, Beijing Institute of Technology, Beijing, China; ^3^School of Psychological Research, Beijing Yiran Sunny Technology Co. Ltd., Beijing, China; ^4^Engineering and Computer Science, University of Denver, Denver, CO, United States; ^5^Beijing Huijia Private School, Beijing, China; ^6^Shouguang Xiandai High School, Shandong, China

**Keywords:** facial expressions, visual mismatch negativity (vMMN), phase lag index (PLI), functional connectivity, gender difference

## Abstract

To investigate gender differences in functional connectivity during the unattended processing of facial expressions, we recorded visual mismatch negativity (vMMN) in 34 adults using a deviant-standard reverse oddball paradigm. Using wavelet analysis, we calculated the time-frequency (TF) power at each electrode associated with happy-deviant, sad-deviant, happy-standard and sad-standard conditions. We also calculated the phase lag index (PLI) between electrode pairs and analyzed the dynamic network topologies of the functional connectivity for happy and sad vMMNs in the delta (0.5–4 Hz), theta (4–8 Hz), alpha (8–13 Hz), beta (13–30 Hz) and gamma (30–45 Hz) bands. The results showed that females induced stronger TF power and PLI values than males in only the alpha band over the whole brain regarding the vMMN. Moreover, females had a higher ratio of the number of connections between long-distance electrode pairs than males. While theoretical analysis of dynamic network topologies indicated that high node degree values were found in local brain regions of males and in almost the entire female brain, our findings suggested that female brain activation and connections between brain regions are not only stronger but also more widely distributed during the unattended processing of facial expressions than those in males.

## Introduction

In the last decade, a large number of studies have been conducted to analyzed emotion processing using electroencephalography (EEG), and many of them evidenced the different processing capacities between the two sexes, showing that females are more competent in detecting feels than males (Schirmer et al., 2002; Campanella et al., 2004; Montagne et al., 2005; Li et al., 2008; Lithari et al., 2010). These studies have found that high-intensity negative emotional stimuli are more likely to irritate people, including males and females, while females are more sensitive to low-intensity negative and positive stimuli, which is unanimously recognized by the public (Campanella et al., 2004; Li et al., 2008; Lithari et al., 2010).

Mismatch negativity (MMN) reveals the differences between event-related potentials (ERPs) elicited by deviant (infrequent) and standard (frequent) stimuli (Tiitinen et al., 1994; Näätänen and Winkler, 1999; Recasens and Uhlhaas, 2017), and reflects the auto-processing of information in the brain without the voluntary participation of the individual. MMN has been widely used in many fields of study such as psychology, cognitive neuroscience and rehabilitation medicine. Such automatic change detection mechanisms operate in the visual modality, as indicated by the visual MMN (vMMN) brain potential to rare changes. Importantly, the vMMN decreased in response to face inversion, indicating the mechanism of high-level-based unattended change detection (Susac et al., 2004; Chang et al., 2010). The vMMN, in response to deviant facial expressions, designates the violation of abstract sequential regularities in facial expressions with a predictive memory representation, but the mechanism of vMMN is not very clear.

In addition, some studies investigated gender differences in unattended processing of vocal stimuli using MMN. Gender differences are found in the lateralization of language. Specifically, MMN amplitudes is significantly larger in females than in males, especially in the right hemisphere, which is estimated during dichotic listening tasks (Ikezawa et al., 2008). The study also revealed that males have left hemisphere and contralateral advantages in scalp-current-density. Nevertheless, there is no significant differences between genders in the MMN values of pure tone tasks. Furthermore, Schirmer et al. (2005) investigated gender differences in the processing of emotional prosody during the unattended stage and found that both sexes exhibited changes. However, only females were influenced by emotional prosody, which was detected using increased EEG amplitudes in MMN. Gender differences have been founded in above researches in sound stimuli in non-attention states, but there are few other studies of gender differences in complex social stimuli, such as facial expressions. In spite of gender differences existing in facial expression processing (Kret and De Gelder, 2012), there is still a lack of evidence for automatic treatment of non-attention situations.

Currently, MMN research is in correspondence to the frequency variable or the time variable, but research involving functional connectivity is rare. In recent years, a great number of studies on the functional brain network have been conducted, showing that the parameters of the network can effectively reflect the various features of the whole-brain network system. Recent studies have shown that emotional processing activates different brain regions, and brain functional connectivity is different between males and females (Tomasi and Volkow, 2012; Zhang et al., 2018). Ingalhalikar et al. (2014) study finds that male brains have more connections within a single hemisphere, but female brains appear to be more connected between the two hemispheres. Functional connectivity studies have reported stronger connectivity in the default mode network in females within the posterior cingulate cortex, et cetera (Bluhm et al., 2008). Stronger intra-network functional connectivity in females or males (Allen et al., 2011) and a mixture of high and low functional connectivities have also been researched (Filippi et al., 2013) in regard to the brain networks. Additionally, activation in different brain network are reported in females and males when people processing emotions of themselves (Schulte-Rüther et al., 2008). However, how functional connectivity affects vMMN males and females is still unclear.

The purpose of this study is to explore gender differences in the unattended processing of facial expressions. We used the deviant-standard reverse oddball paradigm, in which participants engage in a visual detection task presented in the center of the visual field (monitor), while physically identical facial stimuli with different facial expressions were shown in the visual periphery to reduce the influence of low-level physical feature differences between the faces. Then, participants’ vMMN was recorded and compared between the two sexes (Stefanics et al., 2012). In the present study, to minimize the variance associated with the use of genuine facial photographs as stimuli, we used schematic sad, happy and neutral faces as deviants and standard stimuli in different blocks so that the faces themselves were unrelated to the participant’s task. Then, the brain functional connectivity network was constructed from the vMMN time series of males and females to calculate the statistical features of the brain functional network and to study the connections between brain regions. We expected that gender differences in the vMMN might be related to the functional connectivity across brain regions.

## Materials and Methods

### Participants

Thirty-four healthy volunteers (16 females and 18 males) participated in the study, with an age distribution of 23.6 ± 3.2 years for the males and 23.4 ± 1.5 years for the females (mean ± standard deviation (SD)). Thirty-three participants were right-handed, and one female participant was left-handed, as established by the Edinburgh (Oldfield, 1971) handedness questionnaire. Any volunteer who had medication or had a history of psychosis or neuropathy was excluded from the study, and all accepted participants also had normal or corrected visual acuity. To minimize the effects of the personal emotion of the subjects, we have made sure that the participants were in a calm mood. This study was approved by the Ethical Committee of East China Normal University in accordance to the ethical principles of the Declaration of Helsinki. All participants were paid for their participation and gave well-informed written consent prior to the experiment.

### Experiment Settings

The experiment employed three general facial expression sets: happy, sad, and neutral, as the stimuli in the study. Each expression set contains eighteen different schematic facial models with adjusted distance and shape of facial features, particularly the mouth (Xu et al., 2013), hence 54 schematic faces were used in total. A cross that could change in size was presented to the participants as the target stimuli to attract the attention of the participants. The viewing distance was 70 cm, and the visual angle was 3.68° × 3.42°.

**Figure 1:**
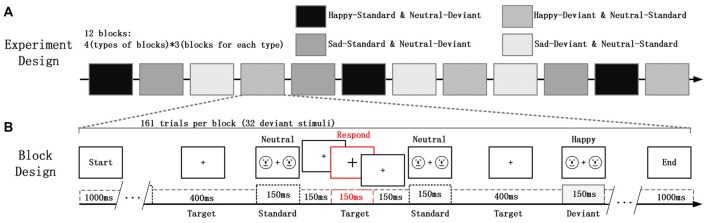
**(A)** The experiment consisted of four types of blocks and a total of 12 experimental blocks (each type of block consisted of three blocks). **(B)** Each block contained 161 trials of two kinds of schematic faces. Two schematic faces displaying the same expression were presented peripherally in each screen for 150 ms. This screen was followed by an inter-stimulus interval of 450 ms, during which the size of the fixation cross was changed occasionally. The participants’ task involved a quick button-press when the cross became larger.

In the study, the deviant-standard-reverse oddball paradigm was used to avoid the impact on vMMN due to physical differences between the emotional faces and the neutral faces; and the Participants were asked to focus on the fixation cross (“+”) whose size was changing irregularly, located in the center of the visual field. At the same time, faces that participants were required to ignore appeared on the right and left sides of the fixation cross (Xu et al., 2013). In this experiment, facial stimuli are presented on the periphery of the participant while the participant needs to perform a visual inspection task presented at the center of the field of view. Participants did not pay attention to facial stimulation throughout the experiment. This task mode has been widely used in vMMN research (Kret and De Gelder, 2012; Stefanics et al., 2012). Schematic emotional faces (sad, happy and neutral) was used as deviants and standard stimuli in different blocks to evaluate the role of faces that are not related to the participant’s task, as shown in Figure 1B. Schematic faces retain low-level physical features and simplify advanced features such as gender races, avoiding excessive information interference and minimizing the hybrid effect of general arousal. Additionally, significantly increased fMRI signal was found in the amygdala, hippocampus, and prefrontal cortex in response to emotional vs. neutral schematic faces, suggesting that, compared to human faces, schematic faces could be a better choice for studying brain responses to emotional stimuli because of their simplicity (Wright et al., 2002). Moreover, the MMN is more sensitive to the deviant feature located on the lower half-field to avoid low-level processing of facial features (Czigler et al., 2004). In addition, some previous studies indicates that even a schematic face made from simple line fragments triggered the face-sensitive N170 and MMN (Sagiv and Bentin, 2001; Kreegipuu et al., 2013; Liu et al., 2013; Xu et al., 2013; Yan et al., 2018). All the facial stimuli were presented with an exposure duration of 150 ms and an inter-stimulus interval of 450 ms, with a pseudo-random selection process in each block. Ten standard stimuli were presented in the beginning of the sequence in order to establish sensory memory patterns. Then, there were no less than two standards between the consecutive deviants. The target stimuli, for which the size of the fixation crosses changed, were always presented without facial stimuli for 150 ms within each interval. The experiment consists of four types of sequences, each containing three blocks of 161 trials (32 trials for deviant stimuli). Using the same face as a bias stimulus and a standard stimulus makes it easier for us to compare the effects of physical stimulation. The influence of low-level physical feature causes less differences between the happy (or sad) faces and the neutral faces in vMMN.

Four types of blocks and three blocks for each type are designed in the experiment as displayed in Figure 1A. The order of the block conditions was counterbalanced across participants. Each participant was seated in a chair in a sound-attenuated and electrically shielded room and asked to ignore the facial stimuli, to focus on the center of the visual field. At the time of the size of cross changing, participants needed to detect it as accurately as possible and respond by key action. They were also required to minimize any eye movement during the experiment. There were several practice trials before the test trials.

### Data Acquisition

Sixty-six-channel EEG data were recorded using a NeuroSky system with a battery-powered amplifier, which was connected to the recording computer (bandpass, 0.05–100 Hz; sampling rate, 500 Hz). The electrode locations were in accordance with the international 10-20 standard system. VEOG and HEOG were recorded with two pairs of electrodes, with one pair placed above and below the right eye and the other 10 mm from the lateral canthi. And electrode impedance was maintained below 5 kΩ throughout the experiment.

### Preprocessing and TF Power Analysis

Our data were preprocessed with Matlab R2014b with the following open source toolbox: EEGLAB^1^. Additionally, an independent component analysis was used to remove artifacts (e.g., eye artifacts, muscle artifacts and electrocardiographic activity) from the data within all channels, with each epoch having their baseline corrected. The EEG data were segmented from the epoch from 300 ms pre-stimulus to 550 ms post-stimulus, which was time-locked to the onset of the faces and included a 100-ms pre-stimulus baseline. The EEG segments were extracted separately for sad and happy faces in different blocks, with an average number of trials of 85, 86, 83 and 85 for sad-deviant, sad-standard, happy-deviant and happy-standard faces, respectively.

To assess brain activity, we averaged the phase-locked spectral power (Stothart and Kazanina, 2013). Epochs were sorted according to stimulus conditions to create a plot of TF representations (TFRs), and the total frequency band responses were analyzed via a complex Morlet wavelet using the Matlab wavelet toolbox (MathWorks, Natick, MA, USA). The wavelet family we used is defined by f0/sf = 7, with f0 ranging from 0.5 Hz to 45 Hz in 0.5-Hz steps. The TFRs of several bands of power were calculated for each participant. These ranged from 1 Hz to 45 Hz in a time window between 300 ms pre-stimulus and 500 ms post-stimulus onset at all the electrodes.

### Functional Connectivity Estimation

In these studies, we performed evoked/event-related power spectra, filtered oscillatory responses, and/or phase locking (inter-trial phase coherence) analyses. Among these methods, the phase locking analysis is one of the most important methodologies that can show functional connectivity between different pairs of electrodes. The functional connectivity between different brain regions may be studied by estimating statistical interdependencies between EEG signals recorded over different brain regions. The phase lag index (PLI), a synchronization measure, reflects the extent of inter-trial phase variability in a given frequency across time (Stam et al., 2007; van Diessen et al., 2013). The PLI is defined as a period of phase locking between two events, and it can only be estimated in a statistical sense. This method of measuring synchronism is attenuated by the near-phase synchronization, which reduces the pseudo-synchronization of homologous signal transmission at one time (Doesburg et al., 2013; Wang et al., 2017).

The non-target stimuli were filtered into the delta (0.5–4 Hz), theta (4–8 Hz), alpha (8–13 Hz), beta (13–30 Hz) and gamma (30–45 Hz) frequency ranges and estimated network synchronization of all four bands. The Hilbert transform was used to obtain the time series of instantaneous phase measures for each trial, source and frequency band. Inter-regional phase locking was calculated for each sensor pair and frequency with the PLI (Wang et al., 2017).

(1) $$\text{PLI = }\left|\langle sign(\Delta \phi (t_{n}))\rangle \right|=\left|\frac{1}{N}\sum_{n=1}^{N}sigh(\Delta \phi (t_{n}))\right|$$

The PLI ranges between 0 and 1. If the PLI is 0, the two signals are either not coupled or are coupled with a phase difference centered at approximately 0 mod π. If the PLI is 1, the two signals are perfectly phase locked at a value of Δφ, which is different from 0 mod π. When this non-zero phase locking is strong, the PLI will be large. However, it is worth noting that the PLI does not indicate which of the two signals is leading in phase.

VEOG, HEOG, M1 and M2 were removed to ensure the reliability of phase relations. Within each frequency band, a sensor-by-sensor adjacency matrix (62 × 62) for each time point was produced. In order to remove the distortion of the edge in the Hilbert transform calculation, the first or last 50 ms (25 sample points) of the epochs are deleted in the part of results. These were averaged within whole-brain areas of each group (females and males) for each trial condition (sad-deviant, sad-standard, happy-deviant and happy-standard). The vMMN component was calculated by subtracting the average PLI values elicited by the standard stimuli from the PLI values of the deviant stimuli. The PLI difference values across sources for each time point reflected the vMMN dynamic network connectivity. A two-sample *t*-test was performed at each time point to compare the differential PLI values of the males and females. Time points with significant gender differences were used to identify windows for further analyses.

### Connection Length Analysis

We counted the number of long- and short-distance connections in the brain network. There were 62 electrodes and 1,981 pairs of undirected connections in the whole brain. We divided all connections into two groups: long connections and short connections. A long connection was a connection length between two nodes that was greater than the average length, and vice versa for a short connection. Each group was sorted according to the value of the PLI. In addition, the number of the first 10%, 10%–20% and 20%–30% was counted and fitted by linear function. Then, based on a weighted cumulative Gaussian distribution, the change trends of long and short connections were analyzed.

(2) $$f(k)=\frac{1}{2}\left[1+erf\left(\frac{k}{\sigma \sqrt{2}}\right)\right]$$

Therefore, the fitting trend of the long connection minus the fitting trend of the short connection was defined as the difference in the change trends (DCT). The simplified formula is as follows:

(3) $$\text{DCT}=\frac{1}{\sqrt{\pi }}\int_{k_{s}}^{k_{l}}e^{-\frac{k^{2}}{2\sigma^{2}}}d_{k}$$

where the parameters k_l_ and k_s_ are the change trends of the number of long connections and of short connections, respectively, with the intensity of the PLI. Then, the parameter σ is 30, which is about twice the standard error of the change trend of the numbers. The DCT indicates the DCT between the long connections and the short connections. The larger the DCT is, the greater the number of long connections with the intensity of the PLI.

### Theoretical Analysis of Dynamic Network Topologies

Functional connectivity among the sensors was measured by computing the PLI for every possible pair during the time window. The results of adjacency matrices were treated as weighted connection graphs. For each trial, within every frequency and participant, one network (62 × 62) was constructed to characterize the functional connectivity during the time course of tasks (Wang et al., 2015). For the constructed brain networks, we calculated the brain network parameter of the degree to examine characteristic variations among the nodes.

### Statistical Analysis

SPSS version 20.0 was used for the statistical analyses. For each frequency, repeated-measures ANOVAs were carried out separately for the TF power and the average PLI values of the time window. The repeated-measures ANOVAs were performed separately to examine the effects of emotion and gender. The Greenhouse-Geisser epsilon value was obtained in all cases in which the repeated-measures data failed the sphericity test. Then, independent-samples *t*-tests were conducted regarding the differences between males and females. All statistical comparisons were two-tailed with *α* = 0.05. Bonferroni correction was used to correct for the effect of multiple comparisons on neural oscillations. Moreover, the results were verified by permutation test.

## Results

### Behavioral Data Analysis

To determine the degree of attention, the accuracy of the target stimuli was evaluated. The evaluation shows no significant group differences or interactions (above 90% for all conditions, *p* values < 0.1) in terms of the accuracy of the target stimuli.

### N170 Components

The grand average ERPs at the occipitotemporal electrode sites elicited by deviant and standard stimuli in both groups (Supplementary Figure S1). Additionally, the statistical reliability of the N170 mean amplitudes (130–210 ms) was tested by one four-way ANOVA of Repeated Measurement data (2*3*2*2) with stimulus type (deviant vs. standard stimuli), hemisphere (left and right) and electrode site (P7/8, PO7/8 and CB1/2) as the within-subject factors and gender as the between-subject factor. For N170 components, the main effects of stimulus type were significant (*F*_(1,33)_ = 15.121, *p* < 0.001 and *F*_(1,33)_ = 6.769, *p* = 0.014 for the happy and sad face condition, respectively). But no main effects for the gender was significant (*p* > 0.1; Supplementary Table S1).

### Phase-Locked Neural Activity

**Figure 2:**
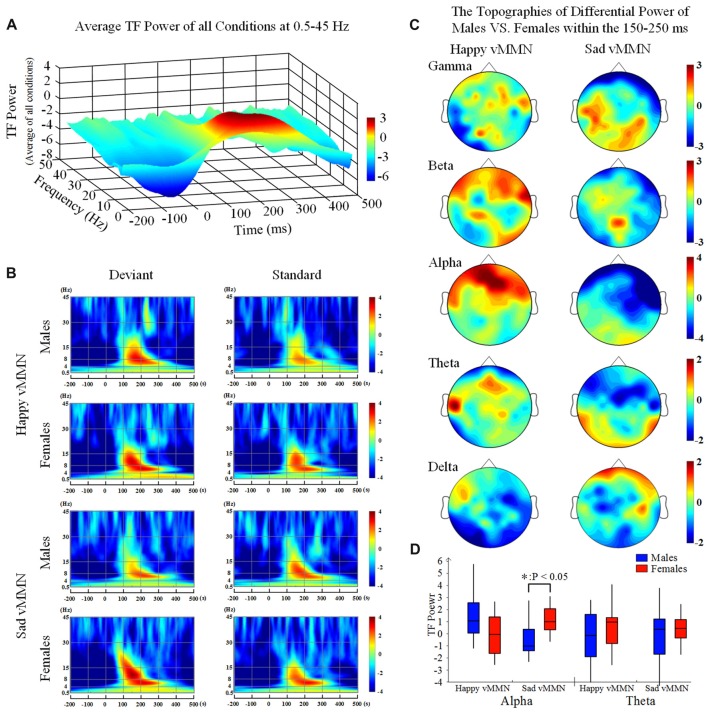
**(A)** The average time-frequency (TF) power at 0.5–40 Hz from −200 ms to 1,000 ms after the onset is illustrated at the all electrodes. **(B)** The TF power shows the phase-locked spectral power at 0.5–45 Hz from −300 ms to 500 ms in response to the happy and sad facial stimuli at all electrodes. **(C)** The topographies illustrate the differential power levels related to males vs. females in a 100-ms time window (from 150 ms to 250 ms) in the all bands. **(D)** A box-plot of the TF power for males and females regarding the happy and sad visual mismatch negativity (vMMN) in terms of the alpha and theta band activity levels during the 150–250-ms time window. *p < 0.05.

Our results showed that both happy faces and sad faces elicited increased evoked power in the theta and alpha bands (Figures 2A,B) within the 150–250 ms after stimulus onset. According to the results of the ANOVAs in the alpha band (Figure 2D), there was a significant interaction of emotion × gender at 150–250 ms (*F*_(2,33)_ = 8.61, *P* < 0.05). In addition, the topographies illustrated that the average activation within the occipital lobe was strong at 150–250 ms in the alpha band (Figure 2C). This indicated that females had greater activation in the alpha band in response to sad stimulation than males did.

### Functional Connectivity

We calculated the PLI for all possible pairs of electrodes for the delta (0.5–4 Hz), theta (4–8 Hz), alpha (8–13 Hz), beta (13–30 Hz) and gamma (30–50 Hz) frequency bands. For each time point, we averaged the PLI of all pairs at each frequency range to acquire the averaged PLI for every frequency band. The functional connectivity of the females was stronger than that of the males in several time segments of the alpha band, but this kind of difference was not shown in the other frequency bands (we do not describe or discuss the results of the functional connectivity in these bands).

#### Time Courses of the Average PLI

**Figure 3:**
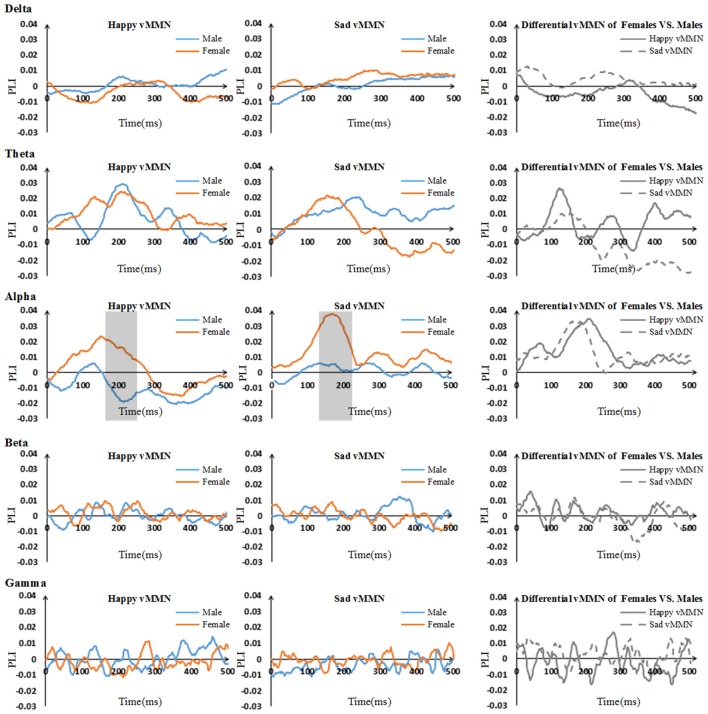
Time courses of the average phase lag index (PLI) values and differential PLI between males and females regarding the vMMN for all participants in the five bands (including 500 ms after stimulus onset). The gray areas are the time windows in which there was a significant difference between males and females.

The results showed that there were clear dynamic changes after stimulus onset for both the happy vMMN and the sad vMMN in the theta and alpha bands, especially for females (Figure 3). The values of the PLI were higher than zero in the alpha and theta bands during a specific period. This finding indicated that the strength of the functional connectivity in females was higher than that in the males in alpha band. However, in the theta, beta and gamma bands, the vMMN network connectivity was inconspicuous.

#### Pairwise Associations Between Groups

**Figure 4:**
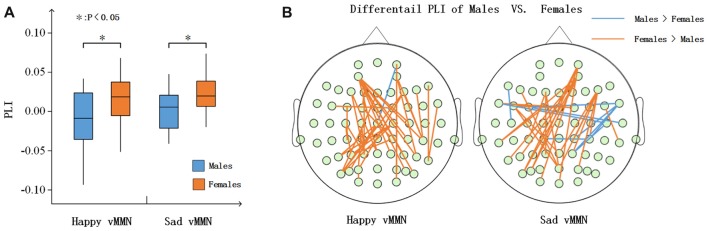
**(A)** Box figure of the average PLI values for males and females in the alpha band. **(B)** Illustration of the difference in the PLI values between the males and the females in the alpha (150–250 ms) band. The brown lines show larger PLI values in the females than in the males, and the blue lines show larger PLI values in the males than in the females (*t*-test was applied and *p* ≤ 0.01). *p < 0.05.

According to the time courses of the averaged PLI, we chose the 150–250-ms time window after stimulus onset to characterize the vMMN-weighted network connectivity. As presented in Figure 4A, we averaged the PLI of the 150–250-ms time window. In the alpha band, there were significant differences between males and females in terms of the happy vMMN (*F*_(1,33)_ = 5.667, *p* = 0.023) and the sad vMMN (*F*_(1,33)_ = 6.634, *p* = 0.015). The *p*-values of permutation test were 0.025 and 0.019 for happy and sad vMMN, respectively. However, there were not significant differences in either of the emotions in the other bands. The detailed results are shown in Figure 4B. The number of strong connections in the females was greater than that in the males.

#### Statistical Results in Task-Dependent Network Topology Parameters via GRETNA

**Figure 5:**
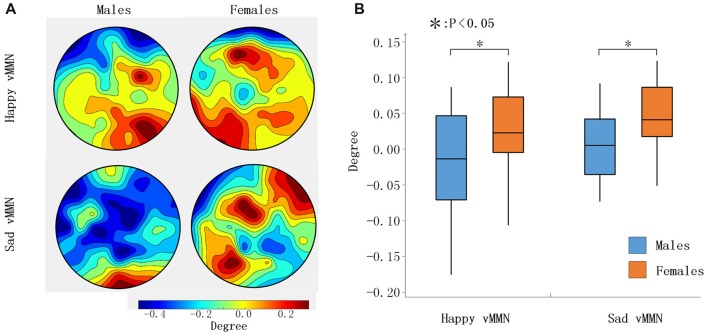
**(A)** The topography shows the distribution of the degree values in regard to the happy and sad vMMN in terms of the alpha band activity at 150–250 ms. **(B)** Box figure of the degree values of the happy and sad vMMN for males and females in the alpha band at 150–250 ms. *p < 0.05.

According to the time courses of the average PLI, we chose the 100-ms time window after stimulus onset (150–250 ms) to characterize the network connectivity. As presented in Figure 3, the PLI values of the female group were larger than those of the male group for the whole brain in the happy and sad vMMN conditions in the alpha band. To assess gender differences in brain network connectivity during the task, the degree of each node was analyzed. As shown in Figure 5A, the topographies were drawn according to the degree of the 62 electrodes of the whole brain, and the results showed that males demonstrated activation of a smaller area than females did. Males exhibited the activation of only the occipital lobe brain regions, and a small area of right middle frontal gyrus was activated during the happy vMMN condition. However, large regions of the occipital lobe, parietal lobe, frontal lobe, middle frontal gyrus and so on revealed significant activations. According to the statistical analyses (Figure 5B), the average degree of the females was significantly higher than that of the males (happy vMMN: *p* = 0.036; sad vMMN: *p* = 0.011). The *p*-values of permutation test were 0.034 and 0.018 for happy and sad vMMN, respectively.

**Figure 6:**
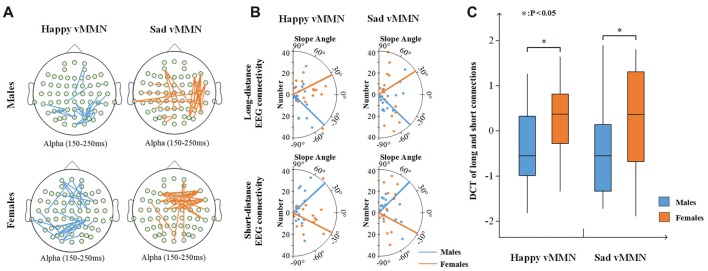
**(A)** The top 25 connections of increased PLI values related to the deviant stimuli compared to the standard stimuli in terms of the alpha (150–250 ms) band activity. **(B)** The number of connections changed with the intensity of the top 30% of the PLI for long- and short-distance electroencephalography (EEG) connectivity. The dot represents the average number of long- or short-distance and the slope angle of the top 30% of the PLI for each participant. The line represents the average slope angle of the top 30% of the PLI for all participants. **(C)** Box figure of the average difference in the change trends (DCT) of the long and short connections for males and females. *p < 0.05.

In addition, the results showed that females had more long connections than males in the happy vMMN and sad vMMN conditions (Figure 6A). For the females (Figure 6B), the number of long connections increased with the increasing strength of the PLI after the calculation, and the number of short connections decreased. In contrast, the males exhibited the opposite trend. A *t*-test of the results (Figure 6C) revealed that there was a significant difference between females and males regarding the DCT for the happy vMMN (*p* = 0.047) and sad vMMN (*p* = 0.048). The *p*-values of permutation test were 0.045 and 0.046 for happy and sad vMMN, respectively. With the increasing strength of the PLI, the percentage of long connections increased for the females, but the percentage of long connections decreased for the males.

## Discussion

Many studies have suggested that females are more sensitive to emotional faces than males are during unattended processing (Schirmer et al., 2002; Campanella et al., 2004; Li et al., 2008, 2018; Lithari et al., 2010). However, the way in which functional connectivity affects the vMMN of different genders is still unclear. To investigate gender differences in unattended processing of facial expressions, we designed an experiment using the oddball paradigm. Our data showed that both happy faces and sad faces elicited increased evoked power and PLI values in the theta and alpha bands within 150–250 ms after stimulus onset. However, the females induced stronger TF power and PLI values than the males did in the whole brain only in the alpha band. Moreover, further analysis confirmed that the gender difference was related to the network structure of the brain’s functional connectivity. Through network structure analysis, the number of connections between long-distance brain regions was found to be greater in the females than in the males during the vMMN, and the nodes with the high degree values were distributed in multiple encephalic regions of the females. The results suggested that females activate a more complicated network in the unattended processing of facial emotion.

The results of ERPs show that the amplitude of deviant were significantly lower than the standard within 130–210 ms in both the happy condition and sad condition, which proves that the vMMN is activated. The results were consistent with previous findings which used schematic faces (Chang et al., 2010; Xu et al., 2013) and natural faces (Zhao and Li, 2006). However, there was no significant difference between males and females for ERPs, consistent with many studies. For instance, the gender differences are found by means of sLORETA but not ERPs amplitude (Xu et al., 2013). In addition, several studies demonstrated that gender differences in processing facial expressions were not conspicuous (Grimshaw et al., 2004; Rahman et al., 2004). Although the gender difference was not found in ERPs amplitude, it was found in global functional connectivity.

In this study, we calculated the PLI of vMMN in a deviant-standard-reverse oddball paradigm. We found that the EEG signal changed in the 150–250 ms range after stimulation. So far, many studies have suggested that the face-sensitive N170 component is modulated by facial emotion (Goffaux et al., 2003; Esslen et al., 2004; Caharel et al., 2005; Bentin et al., 2007; Rossion and Jacques, 2008). One study showed that the unattended mechanism mainly occurs in an earlier period after stimulation, and then the attention and vigilance systems appear later (Posner and Petersen, 1990). An important discovery of this research is that the network connection of the early-vMMN is stronger in female participants than in male participants. In addition, we also find stronger functional connectivity in the theta and alpha bands than in the beta and gamma bands. The previous studies showed that the MMN is related to the theta and alpha bands during emotion processing (Güntekin and Basar, 2007; Gee et al., 2011). It commonly agreed, as the stress of thinking increases, that alpha composition not only increases in an eyes-closed condition but also decreases (Bazanova, 2012). The participants did not focus on the experiment tasks of the vMMN, so the alpha composition was activated during the unattended process. Moreover, the modulation of human EEG activity by facial preference was found in the alpha band power (8–13 Hz) but not in the other frequency bands (Kang et al., 2015). A study also showed differential alpha coherence hemispheric patterns in males and females during pleasant and unpleasant musical emotions (Flores-Gutiérrez et al., 2009). Therefore, gender differences existed in the unattended processes of facial expressions in the alpha band.

Regarding the functional connectivity of the whole brain and the TF analysis, we also inferred that the females were stronger than the males. Previous studies provided a great deal of evidence regarding gender differences in emotional processing (Price, 2017). One neurophysiological study (Lithari et al., 2010) showed that females were more responsive to emotional stimuli. Li et al. (2008) demonstrated the neural mechanism underlying the female advantage in identifying negative emotions. However, our findings showed that more connected areas of the brain were activated in females than in males during unattended change detection of facial expressions in both happy and sad emotion. Schirmer et al. (2005) found that female participants were more sensitive to the emotional valences of facial expressions, a finding that was in line with the present results. The results of our experiments are comparable: according to the PLI, our research finds that female brains had stronger connections between hemispheres. Since some studies suggest that the left hemisphere was mainly closely related to language, consciousness and mathematical analysis, and the right hemisphere was largely related to non-verbal information perception, emotion, music, graphics and the concepts of time and space (Timofti, 2010; Holtgraves and Felton, 2011) and from an anatomical point of view, the corpus callosum of females is wider than that of males, and the rear part of the corpus callosum is the visual cortex (Dubb et al., 2003; Kruggel, 2006). Then, as this region is developed in females, females can analyze and process visual information in a timelier manner. Moreover, one researcher (Ingalhalikar et al., 2014) scanned the brains of 949 young people (428 males and 521 females) using a form of magnetic resonance imaging. This research found that male brains had more connections within hemispheres, but female brains were more connected between hemispheres. The results suggested that male brains may be optimized for motor skills, whereas female brains may be optimized for combining analytical and intuitive thinking. The main function of the corpus callosum is to connect the left and right hemispheres; thus, females have a closer connection between the left and right hemispheres than males do. This makes the functional connection of females stronger than that of males overall. In addition, the regulation of emotion is affected by male hormones. Derntl et al. (2009) examined male participants during the process of emotion recognition and found that testosterone levels affected amygdalar activation, which is one of the important brain areas in the regulation of emotions. According to the studies by Altemus (2006) and Halbreich and Kahn (2007), the volatility of hormones is closely associated with mood disorder susceptibility. The fluctuations in female hormone levels are significantly greater than those of males, which is likely another important cause of the gender differences in emotional processing. With the existing research results, we could suggest that the gender difference in facial emotion processing is present in the stage of unattended processing.

Moreover, the number of connections between long-distance brain regions in females was greater than that in males in vMMN. The processing of emotional pictures is reflected by increased long-distance EEG connectivity (Güntekin et al., 2017). Moreover, in terms of the distribution of the brain topography of the node degree values, the occipital lobe and frontal lobe are the important brain areas of the functional brain network during unattended processing. Studies have shown that the occipital lobe processes visual information, while the frontal lobe activation has strong correlation with the vMMN (Kimura et al., 2012; Xu et al., 2013). Our experiment supports that larger brain regions are activated in females than in males. Moreover, the functional connectivity of the females was stronger between these brain areas during unattended processing. One study showed that females had significantly larger orbitofrontal brain areas (Gur et al., 2002), which is a typical emotional connection area. Hofer et al. (2006) also endorses that females exhibited activation of more brain regions during emotion processing than males. From the illustrated topographies of the TF power and the degree, females exhibited activation in more the brain regions than males did. Moreover, we found that females had more connections between brain areas. One study (Steinmetz et al., 1995) showed that the forebrain volume-adjusted size of the corpus callosum was larger in women than in men. Others (Gur et al., 1999; Gong et al., 2009) investigated sex differences in gray matter and white matter volumes using magnetic resonance imaging technology. The results found that the gray matter volume of females was higher than that in males; thus, females may have more advantages in brain cortex interval connections (Gur et al., 1999; Chen et al., 2007; Perrin et al., 2009; Paus et al., 2010). In conclusion, when dealing with the MMN in regard to facial emotion processing, the brains of females not only have stronger activation but also have more extensive connections. This explains that females achieve sensitive emotional processing by using more brain resources.

There were some limitations to our study. First, we used schematic faces as facial stimuli in order to eliminate the influence of irrelevant information from the faces, including low-level features of the faces and the possible interaction between the genders of the stimuli and of the participants. It is necessary to investigate the hypothesis further using real human faces in order to obtain more confirmative evidence. Second, because both target and non-target stimuli were used as the visual stimulation, mutual interference may have been produced between them. Recently, multisensory stimuli have gained increasing interest, and further studies should use this new method to achieve a better study of facial expression processing.

## Conclusion

This study supports that females induce stronger TF power and PLI values than males in the alpha band over the whole brain. In addition, the number of strong connections between long-distance electrode pairs should be greater in females than in males in the vMMN. From dynamic network topological angle analysis, the index of the Degree and Connection Length showed that the distribution of network connections is wider in females than in males. This suggests that the females enable the whole-brain resources for vMMN, while males induce only local activities. We propose that this gender difference should be considered when researching unattended processing.

## Author Contributions

JZ analyzed and interpreted the data, wrote the article. XD analyzed the data and revised the article. LW and LZ performed the experiments. JW designed the experiments and verified the effectiveness of the experiment. TY provided a schematic of the principle, approved the final version. TZ and JS took part in the work of the initial processing of the data. RG and QH played important role in data processing by providing data processing ideas and processing of the data. RG and QH played important role in data processing by providing data processing ideas and creative methods. ZW made a contribution to the essay writing and modifying the grammar.

## Conflict of Interest Statement

LZ was employed by company Beijing Yiran Sunny Technology Co., Ltd. The remaining authors declare that the research was conducted in the absence of any commercial or financial relationships that could be construed as a potential conflict of interest.

## Footnotes

^1^http://sccn.ucsd.edu/eeglab/

## Abbreviations

DCT: difference in the change trends
EEG: electroencephalography
ERPs: event-related potentials
MMN: mismatch negativity
PLI: phase lag index
SD: standard deviation
TF: time-frequency
vMMN: visual mismatch negativity.
