# Supplementary material for: Gender Differences in Global Functional Connectivity During Facial Emotion Processing: A Visual MMN Study
Source: Front Behav Neurosci. 2018 Sep 25;12:220. doi: 10.3389/fnbeh.2018.00220 (PMC6167960; doi:10.3389/fnbeh.2018.00220)
Supplement: Supplementary file 1 [file Data_Sheet_1.docx]

**Supplementary material**

Supplementary Figure 1


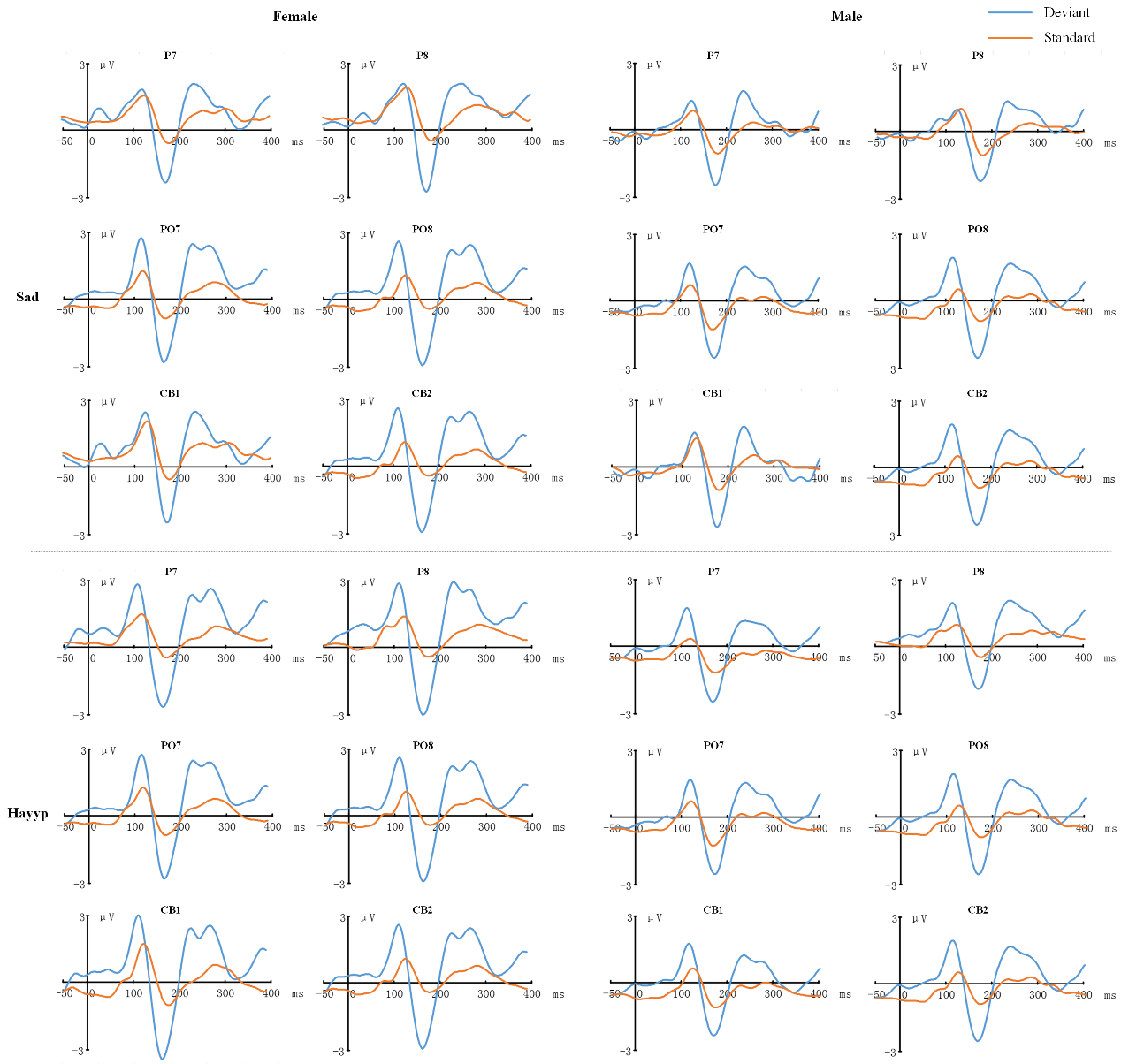


The grand-average ERPs elicited by standard and deviant face expressions at P7/8, PO7/8, and CB1/2 sites.

The statistical reliability of the N170 mean amplitudes (130–210 ms) was tested by one four-way ANOVA of Repeated Measurement data (2*3*2*2): stimulus type (deviant vs. standard stimuli), hemisphere (left and right) and electrode site (P7/8, PO7/8, and CB1/2) as the within-subject factors and gender as the between-subject factor.

For the happy condition, the main effects of sex in tests of between-subjects effects are no significant (F(1,33)=0.003 p = 0.955) for N170 amplitudes. As for sad condition, the main effects of sex in tests of between-subjects effects are no significant (F(1,33)=0.062 p = 0.804). As for test of within-subjects, all results displayed in the table. The results of significant differences are represented in bold fonts. As for test of within-subjects, all results displayed in the table. The results of significant differences are represented in bold fonts.

within-subject effects

| Effect | Happy Condition | Sad Condition |
| --- | --- | --- |
| Site | **F(2,66)=19.327**  **P<0.001** | **F(2,66)=11.046**  **P<0.001** |
| Site*Gender | F(2,66)=2.312  P=0.118 | F(2,66)=1.353  P=0.264 |
| Hemisphere | **F(1,33)=4.633**  **P=0.039** | F(1,33)=3.331  P=0.077 |
| Hemisphere*Gender | F(1,33)=0.068  P=0.796 | F(1,33)=0.006  P=0.941 |
| Stimulus | **F(1,33)=15.121**  **P<0.001** | **F(1,33)=6.769**  **P=0.014** |
| Stimulus*Gender | F(1,33)=0.321  P=0.575 | F(1,33)=0.016  P=0.900 |
| Site* Hemisphere | F(2,66)=2.868  P=0.064 | F(2,66)=3.479  P=0.054 |
| Site*Hemisphere *Gender | F(2,66)=0.401  P=0.672 | F(2,66)=0.407  P=0.598 |
| Site* Stimulus | F(2,66)=2.370  P=0.114 | F(2,66)=1.428  P=0.247 |
| Site*Stimulus *Gender | F(2,66)=0.314  P=0.684 | F(2,66)=0.286  P=0.692 |
| Hemisphere* Stimulus | F(1,33)=2.952  P=0.095 | F(1,33)=1.447  P=0.238 |
| Hemisphere* Stimulus *Gender | F(1,33)=0.001  P=0.972 | F(1,33)=0.011  P=0.917 |
| Site*Hemisphere* Stimulus | F(2,66)=2.921  P=0.076 | F(2,66)=0.2.409  P=0.116 |
| Site*Hemisphere* Stimulus *Gender | F(2,66)=0.112  P=0.841 | F(2,66)=0.179  P=0.729 |

NOTE:

Mauchly's Test of Sphericity was executed to test whether the results need to be corrected. For the happy condition, effects of Site(W = 0.792, p = 0.027), Hemisphere (W = 1.00, p<0.001), Stimulus(W = 1.00, p<0.001), Site*Stimulus (W = 0.754, p = 0.013),Hemisphere* Stimulus (W = 1.00,p <0.001), and Site*Hemisphere* Stimulus(W = 0.692 p = 0.003) are corrected by Greenhouse-Geisser in the tests of within-subjects. The effect of Site* Hemisphere (W= 0.857, p = 0.091) was sphericity assumed, without corrected. For the sad condition, effects of Site(W = 0.706 p = 0.004), Hemisphere (W = 1.00, p<0.001), Stimulus(W = 1.00, p<0.001), Site* Hemisphere (W = 0.586 p < 0.001), Site*Stimulus (W = 0.686 p = 0.003),Hemisphere* Stimulus (W = 1.00,p <0.001), and Site*Hemisphere* Stimulus(W = 0.391 p < 0.001) are corrected by Greenhouse-Geisser in the tests of within-subjects.
